# MiniCERNBot Educational Platform: Antimatter Factory Mock-up Missions for Problem-Solving STEM Learning

**DOI:** 10.3390/s21041398

**Published:** 2021-02-17

**Authors:** Josep Marín Garcés, Carlos Veiga Almagro, Giacomo Lunghi, Mario Di Castro, Luca Rosario Buonocore, Raúl Marín Prades, Alessandro Masi

**Affiliations:** 1 CERN, BE-CEM Controls, Electronics and Mechatronics Group, 1217 Geneva, Switzerland; josepmaringarces@gmail.com (J.M.G.); carlos.veiga.almagro@cern.ch (C.V.A.); mario.di.castro@cern.ch (M.D.C.); luca.rosario.buonocore@cern.ch (L.R.B.); alessandro.masi@cern.ch (A.M.); 2 Interactive Robotic Systems Lab, Jaume I University of Castellón, 12006 Castellón de la Plana, Spain; giacomo.lunghi@cern.ch

**Keywords:** sensors, robotics, mechatronics, physical devices, education, STEM education, interaction, engineering education

## Abstract

Mechatronics and robotics appeared particularly effective in students’ education, allowing them to create non-traditional solutions in STEM disciplines, which have a direct impact and interaction with the world surrounding them. This paper presents the current state of the MiniCERNBot Educational Robotic platform for high-school and university students. The robot provides a comprehensive educative system with tutorials and tasks tuned for different ages on 3D design, mechanical assembly, control, programming, planning, and operation. The system is inspired to existing robotic systems and typical robotic interventions performed at CERN, and includes an education mock-up that follows the example of a previous real operation performed in CERN’s Antimatter Factory. The paper describes the learning paths where the MiniCERNBot platform can be used by students, at different ages and disciplines. In addition, it describes the software and hardware architecture, presenting results on modularity and network performance during education exercises. In summary, the objective of the study is improving the way STEM educational and dissemination activities at CERN Robotics Lab are performed, as well as their possible synergies with other education institutions, such as High-Schools and Universities, improving the learning collaborative process and inspiring students interested in technical studies. To this end, a new educational robotic platform has been designed, inspired on real scientific operations, which allows the students practice multidisciplinary STEM skills in a collaborative problem-solving way, while increasing their motivation and comprehension of the research activities.

## 1. Introduction

Mechatronics and robotics play an important role in high-school and University education. They provide the students with real world challenges to be solved using novel solutions and pushing into a deeper understanding and direct application of Science, Technology, Engineering, and Mathematics (STEM) language and systems. STEM education aims to provide the knowledge and tools to the widest number of students for pursuing careers in STEM fields [1]. Mechatronics and robotics provide access to all STEM fields by showing how they work in real life and revealing their impact in the world of tomorrow.

Education is an important mandate for CERN, whose role is to educate Europe’s future scientists and engineers and provides a series of educational programmes targeting students of different ages [2,3]. Of the 100,000 visitors who visit CERN each year, the majority are high-school students [4,5]. Opportunities for students in applied physics, engineering, computing, and more are available throughout the whole year, thanks to workshops and internships like high-school internship programme, the Beamline for School challenge, and the S’Cool Lab research facility [6].

The Mechatronics, Robotics and Operations (MRO) section at CERN, part of the Survey, Mechatronics and Measurements (SMM) group of the engineering department is in charge of designing and developing cutting-edge robotics technology to remotely perform real interventions in the accelerator scientific facilities. The CERNBot and Train Inspection Monorail (TIM) platforms are an example of such systems, which are continuously improved and adapted to the CERN necessities, while offering new scientific improvements to the Telerobotics research community [7,8,9,10,11]. The MRO section is part of different CERN’s educational programmes and hosts periodically students between the ages of 16 and 19, from diverse background and education. In addition, Bachelor and Master’s students take part in longer education activities, related to more specific scientific problems, which need basic training on STEM related skills. Besides this, in collaboration with Universities, CERN offers Doctorate Programs, which might need desktop educational kits for prototyping and preliminary scientific experiments.

STEM is an approach focused on the development of skills in multiple fields during learning. The use of robotics in STEM (Educational Robotics) can increase students’ interest and motivation [12], both during the learning process as well as future career decisions [13,14,15]. Several studies have been done to present the impact of Educational Robotics (ER) even in young students [16,17,18], thanks to its connection with play and enjoyment is considered to be an important factor that encourages children and enables intrinsic motivation, especially in primary education [19]. Researchers have attempted also to create robotic curricula in high-schools and studying their effect [20,21]. In addition, over the last decade, the interest and engagement of teachers and professor around Educational Robotics and STEM have increased [22]. Different types of educational robots have been presented, adapting to different programs, some more software-centred, others more focused on hardware and mechanical assembly, others on socialisation, interaction and gaming [16]. Overall, Educational Robotics pushes the students at all ages to provide innovative solutions to real-world problems, promoting collaborative skills [23], creativity and problem-based learning [24] and allowing them to learn with technology rather than learning about it [25].

In this paper, the MiniCERNBot Educational Robotic kit is presented. The robotic kit brings the experience obtained during CERN’s robotic operation to the educational level. The robotic kit includes different learning paths adapted to different students’ ages and different training times, spanning from one day to a couple of months. According to their age and skills, students are required to analyse and understand the requirements of a robotic intervention, inspired by real operations performed in CERN’s accelerator complex. They can define the intervention procedures including checklists, failure analysis and recovery scenarios as defined by the CERNTAURO framework for remote interventions [8]. The students can design and manufacture custom tools to simplify the operations’ tasks, program a control application for the robot and additional behaviours on the robot control board thanks to the full support of the CERN Robotic Framework [7] and then apply their work to the real robotic intervention. Finally, they can compare their work by controlling the MiniCERNBot educational robot with the same Human–Robot Interface used to control the CERN’s robots.

### Research Method and Paper Structure

The paper faces the need of a new educational procedure at CERN Robotics Lab, which is centred on solving real related problems, can be adapted to different educational levels (i.e., high-school, Bachelor’s and Master’s), is flexible in terms of duration of the activity (i.e., from one day to three months), allowing collaborative multidisciplinary teams, and greatly helping students to clarify their vocations in STEM carriers.

In order to walk in this direction, the research has focused on three main aspects: (1) The design of a multidisciplinary and adaptable learning path oriented on solving missions of increasing difficulty, (2) the design of a mechatronic educational kit (i.e., MINICERNBot twins and antimatter factory panel) in order to be used as learning scenario and validation tool, and (3) assessment of the performance, in terms of both mechatronics, and final motivation of the students. The education experiments that provided results on students motivation have been performed with high-school, fourth year Computer Science Engineering, and first year Robotics&AI Master’s students.

With the aim of helping on the repeatability of the experiments, the paper proposes a multidisciplinary education path, providing a model to combine STEM practices in the same learning process, identifying free tools that can help enormously in this process. The learning path can be adapted to other education STEM scenarios as required. Besides this, the robotic kit is explained in detail, focusing specially on the used electronics and computer science tools, as well as evaluating their performance by using standard benchmarks (i.e., Android and PC).

To this aim, the paper presents first the proposed learning paths, highlighting the differences and possible customisation according to the student’s age and internship duration. Afterwards, the technical details of the educational kit, both from the mechanical and software point of view, are explained in detail. Finally, the results and video demonstration are shown, including a resume of the experience of various student groups.

## 2. Learning Paths

The education activity presented in this paper follows a collaborative problem-solving learning strategy [26], where the students are introduced to the problem by on-site experts’ (e.g., recovering a radioactive source), study the available tools (e.g., robotic platforms, 3D printers, programming), and develop a solution, which has to be designed, implemented, tested, and applied in a simplified and safe mock-up of the radioactive scenario.

The different teams can solve the problem in a synchronised or decoupled way, getting quantitative feedback from the experts on the efficiency and quality of the solution, according to the tasks that were successfully completed, and being able to compare their achievements with respect to previous teams.

**Figure 1:**
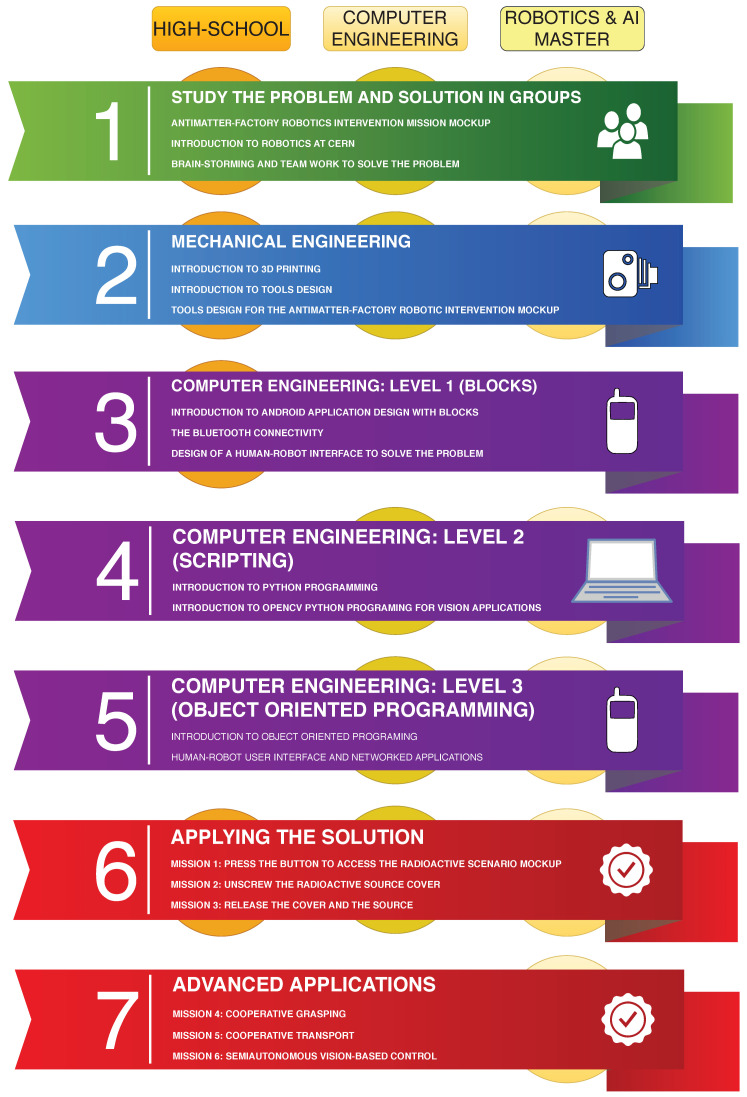
Learning activities using the MiniCernbot platform.

**Figure 2:**
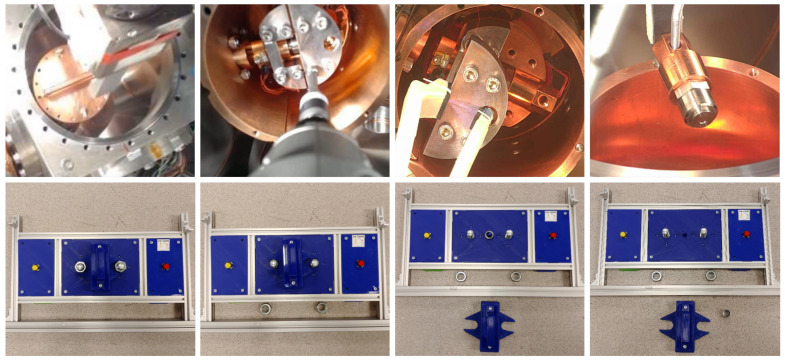
(First row) Antimatter factory real intervention performed by the CERN-EN-SMM-MRO team to recover a radioactive source; (Second row) Educational Mockup simulating the necessary steps to recover a source.

Students visiting the CERN-EN-SMM-MRO Robotics Lab are divided in teams and are offered different learning paths according to their education level (Figure 1). Independently from their age, the teams are required to solve a set of missions on the antimatter mock-up (Figure 2), using different appropriate tools adapted to their educational background.

It is worth mentioning that CERN offers education activities for primary school (e.g., demonstrations and labs), high-school (i.e., summer education program), Bachelor’s (e.g., final project and summer internships), Master’s (e.g., final master project), and Doctorate levels (i.e., collaborating with Universities). Bringing recent discoveries to society via education and dissemination is one of the goals of the institution. The MINICERNBot platform has been designed to help in this task, focusing, at the first stage, on High-School, Computer Engineering and a Master’s on Robotics&Artificial Intelligence students, as can be appreciated in the current Learning selected Paths (see Figure 1). The selected education levels try to demonstrate that the system offers students a tool to develop knowledge based on incremental and multidisciplinary problems, motivated by real experiences. High school students need a tool to discern a vocation, showing both the problems and the possible solutions. Engineering and Master’s students need the tool to better develop their technical capabilities, as well as enhancing their motivation in the subject.

### 2.1. Study the Problem and Solution in Groups

The education activity starts with the introduction to the problem, where scientific experiments must be performed in radioactive scenarios, which do not allow humans to directly interact with the equipment and tools.

As an example of such activities, the students are introduced to a previous experiment performed in the Antimatter Factory, where a radioactive target had to be replaced. In fact, the students are provided with the simplified Antimatter Factory Mockup, as shown in Figure 2, where they are invited to reproduce the real intervention in more simple way.

**Figure 3:**
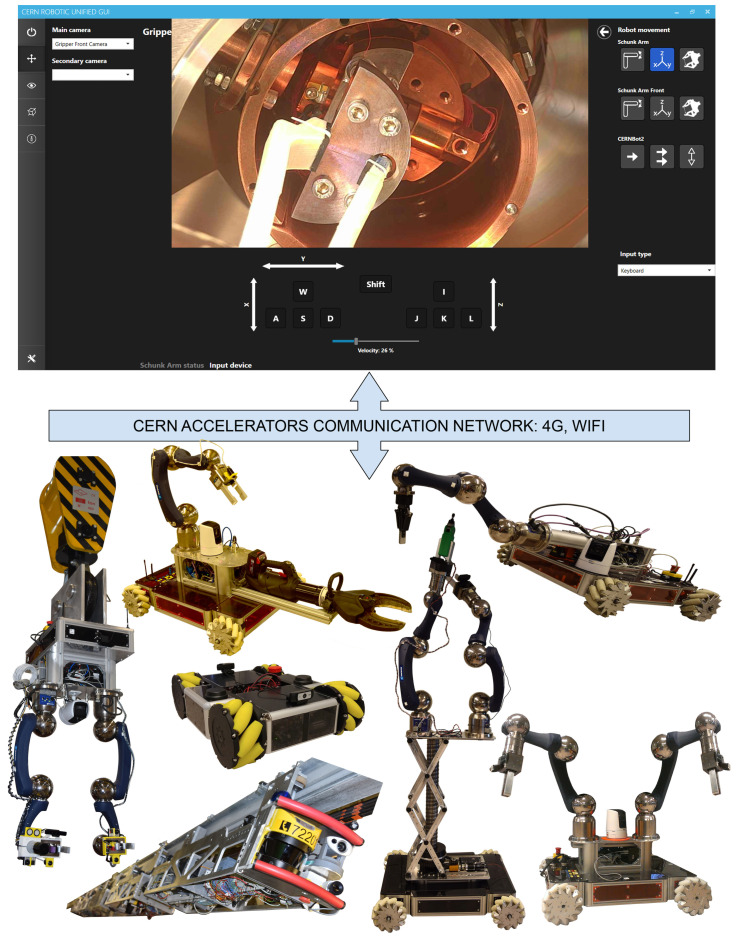
Set of modular robots developed at CERN-EN-SMM-MRO section to perform safe operations in radioactive and hazardous scientific facilities (i.e., Cranebot, Charmbot, CERNBot, CERNBot2, TIM, and Unified GUI).

The second step is the introduction to the robotic facilities at CERN, where the students can face real demonstrations of the modular robots, such as the TIM, CERNBot, CERNBot2, Cranebot, Charmbot, etc., (see Figure 3). In addition, they can better understand the way the robots are remotely operated via the Unified Graphical User Interface (GUI [7]), which guarantees human safety by avoiding their exposure to radioactive scenarios. In addition, the students are able to better understand the meaning of <<Science for Peace>>, focusing exclusively on research and applications that have an outstanding social benefit (e.g., medical applications).

**Figure 4:**
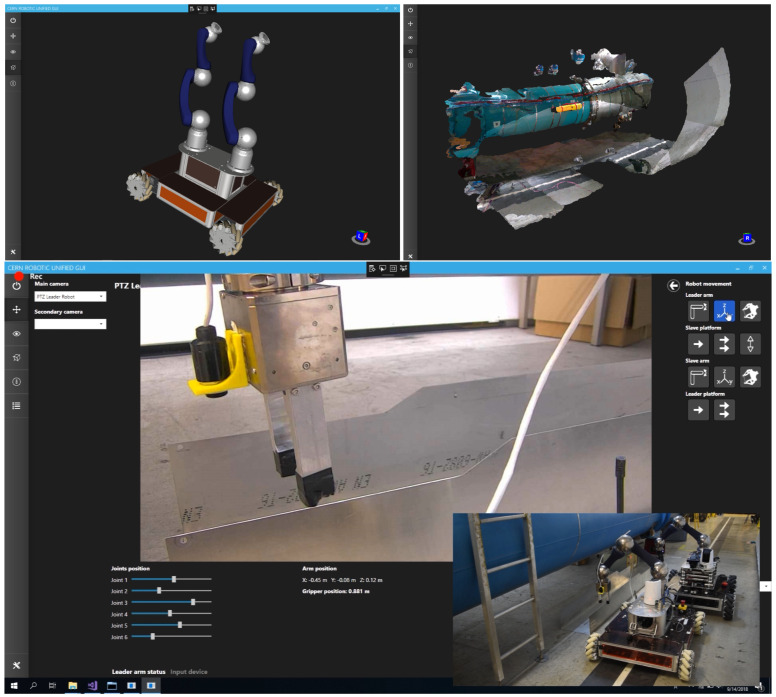
Unified Graphical User Interface to let the operator remotely control the robots in the accelerators and scientific facilities.

The main contribution of this pool of robots is their modularity, which permits adapting the robot configuration to the specific operation to be performed. New tools have to be designed and assembled for each specific intervention, which needs a continuous development and improvement of the robot platforms. In addition, the software used by the operators to control the robots is also explained, so that they can better understand the different ways to interact with the robots, from manual to supervisory control, according to the specific necessity and the expertise of the operator (see Figure 4).

**Figure 5:**
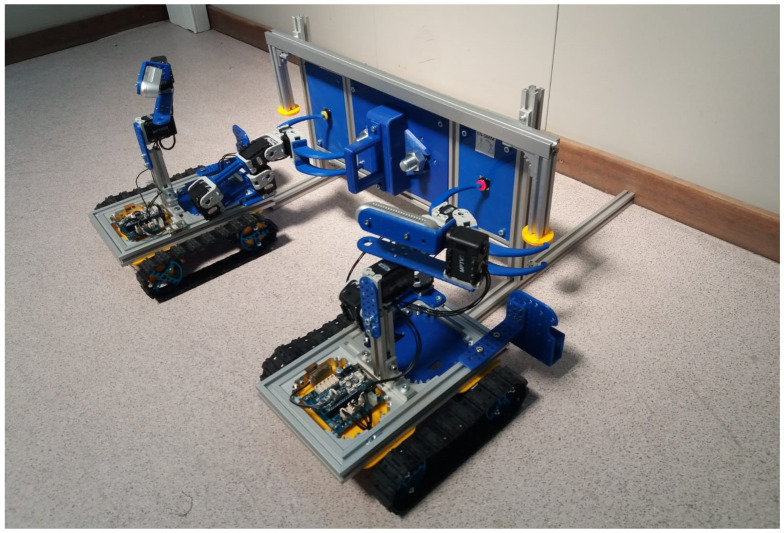
The MiniCernbot platforms and antimatter factory intervention panel mockup.

Once the students understand the robotic platforms, adapted tools, and user interface, the Minicernbot educational robots are explained in detail (see Figure 5). As will be seen later in this paper, the new educational robot is inspired by the CERNBot one, and enables the student to reconfigure the position and number of robotic arms, as well as the camera head. In addition, it allows the students to use tracks or omnidirectional wheels, according to their specific necessities. The robot can be also enhanced mechanically by the students by attaching tools and components to the gripper and aluminium frame structure, according to their specific solutions.

The next step in this first learning group (i.e., <<Study the Problem and Solution in Groups>>) is a comprehension of the problem to be solved. According to a previous real intervention in the radioactive Antimatter Factory, the robot had to disassemble a cover and replace a radioactive source. This inspired realistic scenario was simplified for education, so the <<Antimatter Factory Mockup Panel>> was designed (see Figure 2).

The students are required to organise themselves in multidisciplinary groups and face the development of a software and mechatronic solution, based on the Minicernbot platform, to solve the following missions:

- *Mission 1: Press Red Button*. The robot has to approach the Panel, from the base station, and press the red button, which stops the machine, in order to allow the robot to disassemble it.
- *Mission 2: Unscrew the Radioactive Source Cover*. The robot has to unscrew two nuts that are holding the cover of the radioactive source. If the nuts are brought to the base, the mission gets extra points.
- *Mission 3: Uncover the Pretended-Radioactive Source*. Once the nuts are released, the robot has to uncover the radioactive source by grasping the handle of the cover. If the cover is brought to the base, extra points are assigned to the team.
- *Mission 4: Release the Pretended-Radioactive Source*. The team is asked to, once the cover is removed, grasp the pretended-radioactive source and release it from the holder. If the pretended-radioactive source is brought to the base, extra points are assigned to the team.
- *Mission 5: Press Yellow Button*. The robot has to press the yellow button to set up the machine.

The missions proposed have demonstrated to be accessible for the students and also challenging. For further educational experiments, or longer projects, they can be updated by letting the students replace the pretended-radioactive source by a new one, cover it, and screw the nuts. This would require much more effort to design the solution.

The panel also includes a long pipe aluminium frame with two handles, which has been provided in order to let the students recover it to the base by using two Minicernbot platforms, in a cooperative way. This mission has been reserved as an extra exercise.

### 2.2. Mechanical Engineering

Once the students understand the problem to be solved on the panel (e.g., unscrew, remove the plate, recover the pretended-radioactive source, etc.), and the team has been organised appropriately, they are given an introduction to 3D design and printing, in order to provide their own solutions of tools used to perform the operation on the panel.

**Figure 6:**
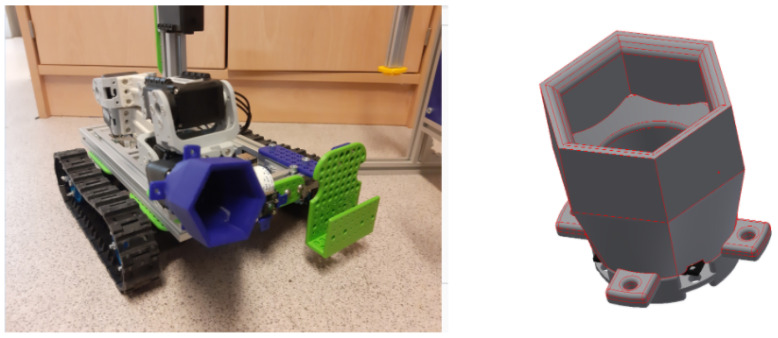
Example of tool designed by a high-school team during a stage at the Robotics Lab.

In Figure 6, an example of a tool designed by high-school students to be able to solve the Mission 2 (i.e., Unscrew the cover of the pretended-radioactive source) can be appreciated.

**Figure 7:**
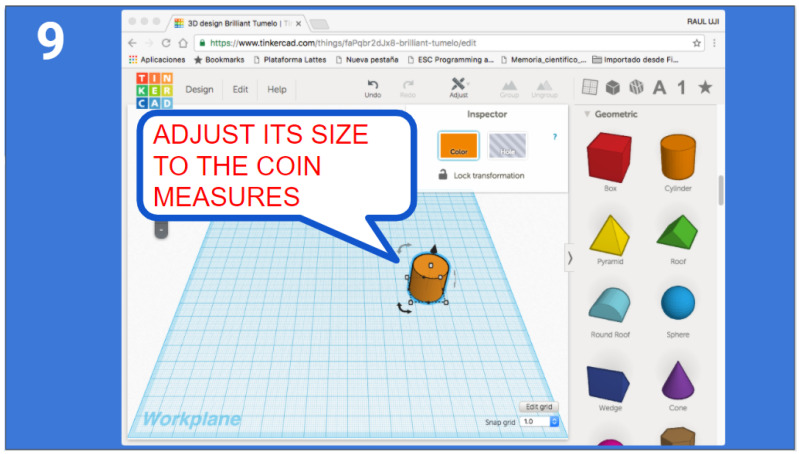
Example of orientations given to the high-school students to introduce the 3D tools design.

The high-school students get a step-by-step guidance in order to get introduced to 3D design and printing. At the moment of writing this article, the tool that has been used for high-school teams is Tinkercad [27], which enables them to create accurate designs by using primitive shapes and operations via a single web interface (see Figure 7).

**Figure 8:**
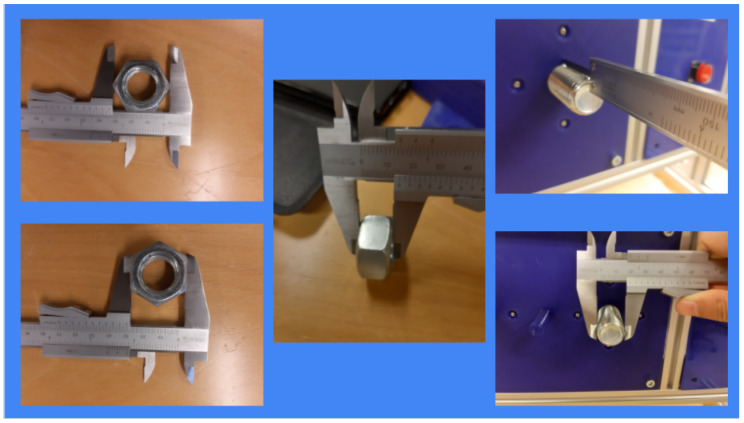
Students measuring the panel in order to design the tool to accomplish the Unscrew Mission.

**Figure 9:**
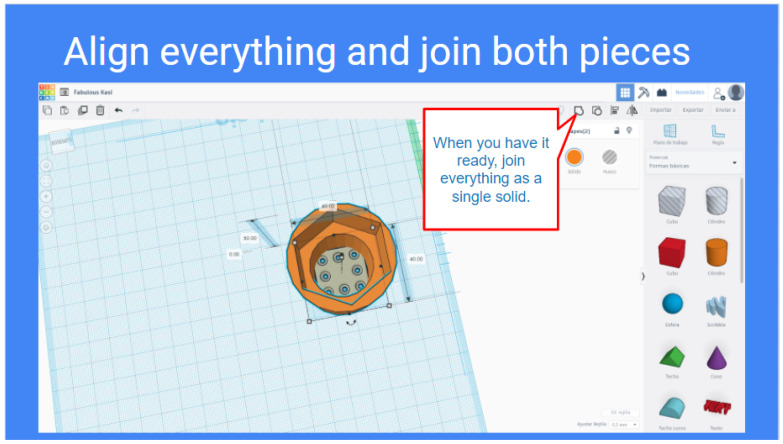
Tool designed by students to unscrew.

Tinkercad is a free, online and easy to use app used to teach the basic concepts of 3D designing. The students are provided with a step-by-step presentation that helps them create their first 3D design, and explore the Tinkercad platform. Once they are confident on 3D design, they are encouraged to proceed with the tool design for the MiniCERNBot, and reminded to be creative when approaching the task (see Figure 8 and Figure 9). Different orientations and examples of real solutions are provided, and for the students that are struggling to accomplish the task, an already designed base piece is provided with exact measurements for the attachment with the robot’s motor, which is normally the area where most common errors occur.

**Figure 10:**
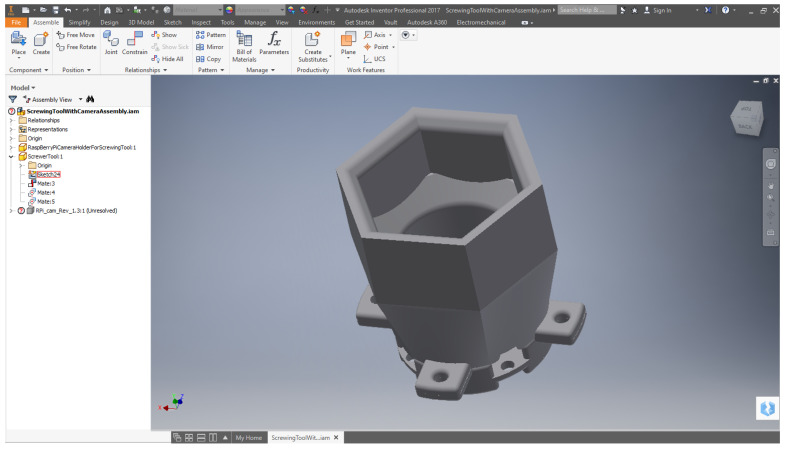
Example of tool designed by advanced students using Autodesk Inventor.

**Figure 11:**
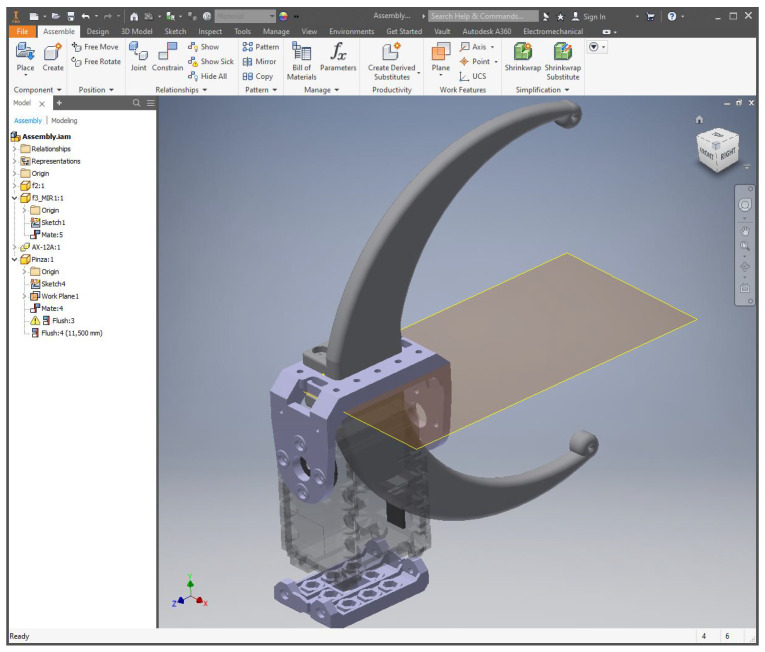
Example of orientations given to Bachelor’s and Master’s students to design a gripper.

The Bachelor’s and Master’s students and given a tutorial on advanced 3D design with parametric tools. At the moment of writing, the tool used is Autodesk Inventor [28], as can be appreciated in Figure 10 and Figure 11. This is a tool that permits more advanced designs, using both primitives and parametric extrusions based on 2D sketches, as well as assemblies, joints, and renderings, among others. The students are given a detailed tutorial on the way advanced parts are designed and manufactured using this tool.

**Figure 12:**
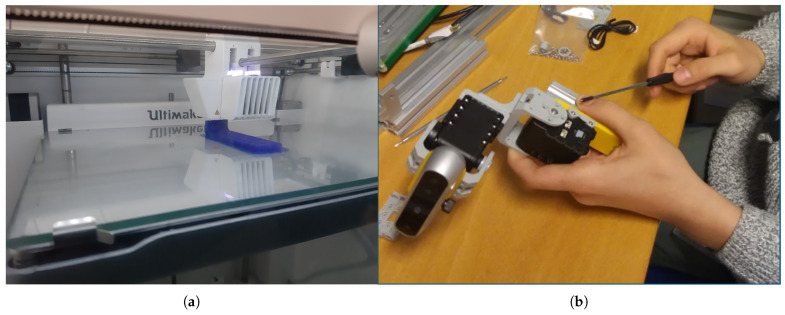
Students assembling their printed tool: The students also learn about how 3D printers work as they print their piece (**a**) printing tools in 3D; (**b**) assembling the tool in the robot to solve the missions.

Once the designed tools have been reviewed, they are printed in plastic and assembled in the robot by the students for real validation, as shown in Figure 12.

When they have finished the design, they are taught how to export their design in 3D format so that they can print it with a 3D printer, and, once printed, they learn how to assemble it into the robot with bolts and nuts previously provided, in order to get the robot ready for the intervention (see Figure 12).

### 2.3. Computer Engineering Level 1 (Blocks)

**Figure 13:**
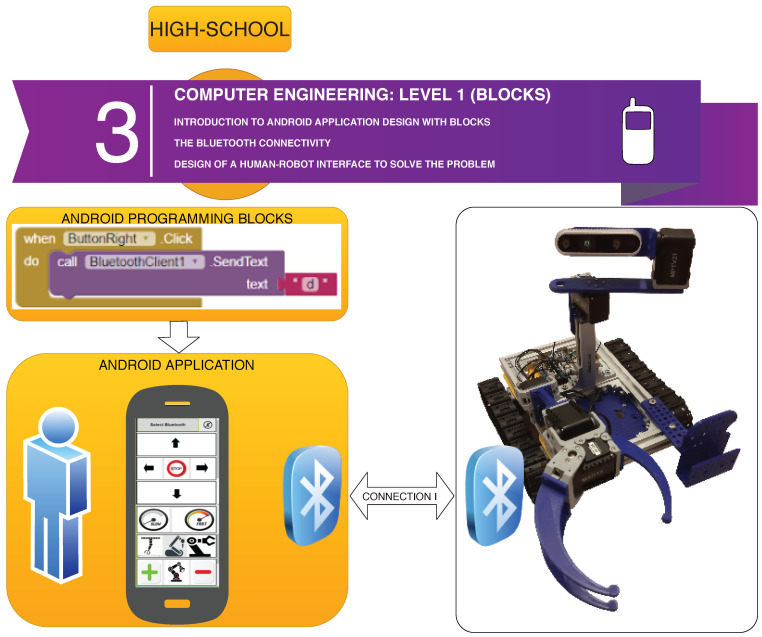
Software architecture for the Computer Engineering Level 1 exercise.

**Figure 14:**
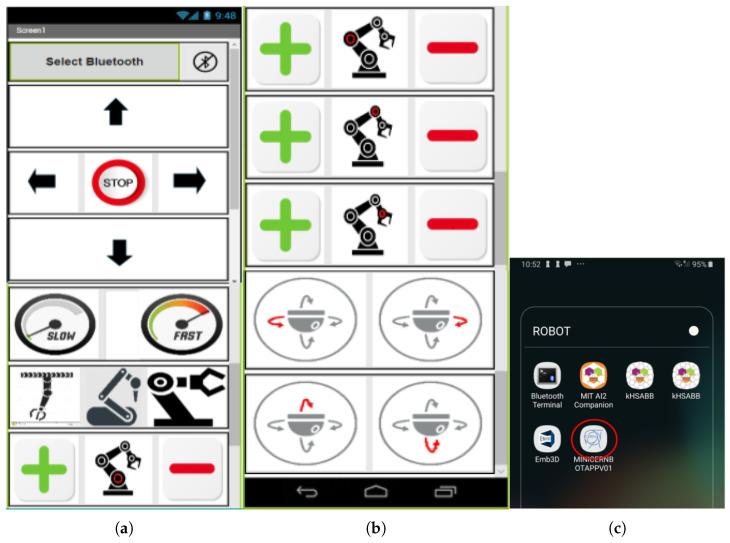
Example of Android User interface created by a high-school student (**a**) base controls; (**b**) arm controls; (**c**) Android Application Logo defined by the student.

In Figure 13, the software architecture for the Computer Engineering Level 1 exercise is shown. The students are given a tutorial on Android Programming by using the MIT App Inventor Tool, which is based on the use of simple programming blocks. The application they develop (see Figure 14) establishes a Bluetooth connection to the robot sensors/actuators controller, so that the students learn how to solve the robot missions by having a direct field of view to the robot from the Android User Interface, so no camera feedback is required in this exercise.

**Figure 15:**
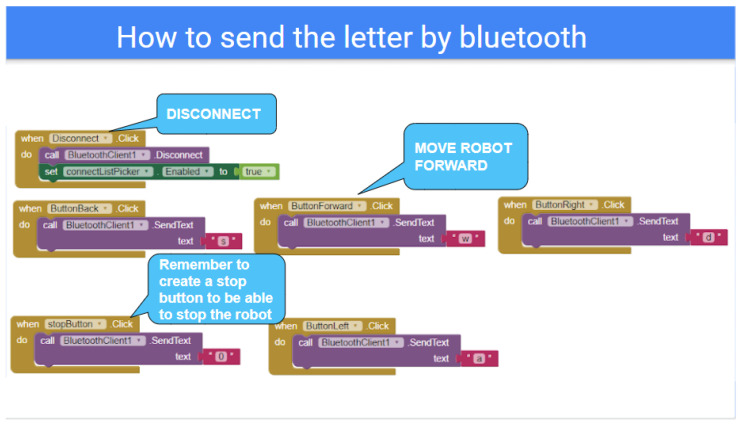
Example of blocks programmed by a high-school student to move the robot platform via Bluetooth).

**Figure 16:**
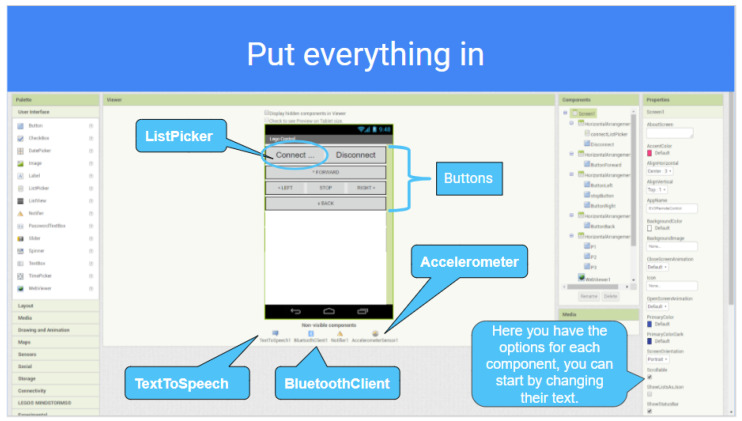
Example of tutorial given to the high-school students to learn how to design a User Interface.

In order to design the first Human–Robot Interface, the MIT App Inventor tool is used by the students to practice Block-Programming (see Figure 15). This gives them a quick way to get the robot working and solve the simple operations. With the help of the step-by-step tutorial they are provided with, the students start to add buttons and other components to their app (see Figure 16). Afterwards, they start programming each component individually with different blocks that they can attach together. The MiniCERNBot robotic platform already has a bluetooth server firmware running on its micro-controller, which starts running as soon as the robot is turned on. This program makes the robot move whenever the robot is sent a character via Bluetooth. This makes programming much more simple as the students only need to connect to the robot and send one of the characters that are provided to them to start the movements. Once they have programmed the basic buttons (forward, backwards, left, right), they already understand what is going on in their app; therefore, they are given different optional tasks to improve their human–robot user interface such as:

1. Adding images to the buttons.
2. Using the Android phone accelerometer sensor to send movements.
3. Controlling the speed.
4. Improving the control.
5. Controlling the robot with the orientation sensor.
6. Optionally, for advanced high-school students, they are given a second Android phone with an HTTP camera application, which provides camera feedback via Wifi, so they can add an HTTP client controller to their blocks program, in order to obtain the robot images on the user interface.

### 2.4. Computer Engineering Level 2 (Scripts)

Engineering and Master’s students are offered a more advanced tutorial on programming languages, letting them use Python in order to design their own robot user interface to solve the antimatter factory mock-up missions.

In this case, the required distance between the student (i.e., operator) and the robot environment is bigger, so the student does not have a direct view of the robot side. The information has to come from the robot sensors, including the cameras.

**Figure 17:**
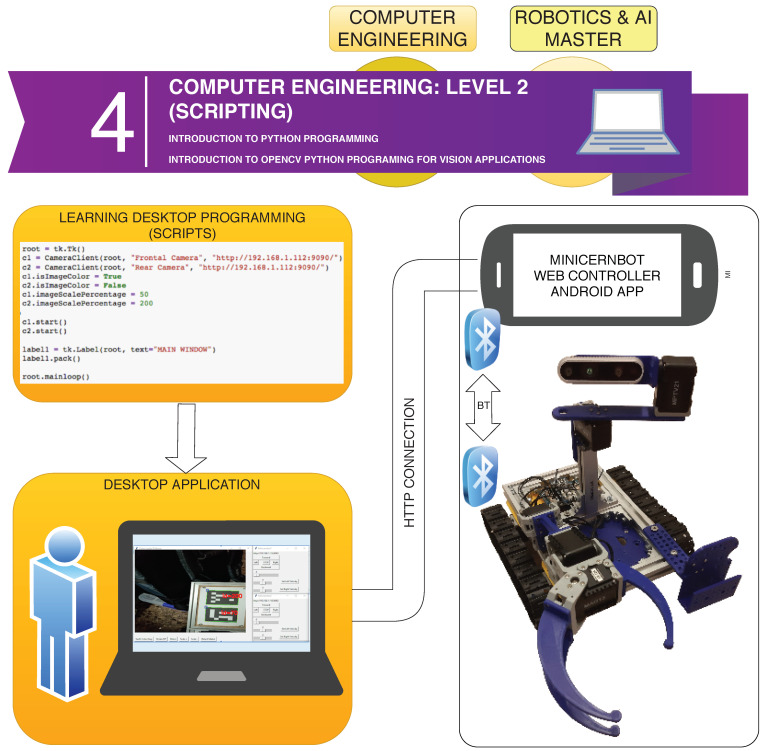
Software architecture for the Computer Engineering Level 2 exercise (Scripts).

As can be seen in Figure 17, for this exercise, the students are provided with an Android Application designed at the Jaume I University (i.e., RobotCamWeb), which connects to the robot via Bluetooth, and offers a Web interface (i.e., HTTP) to both the mobile phone sensors (e.g., Cameras) and the robot motors’ control. The Android Phone is attached to the robot, so that the cameras offer a good point of view of the intervention.

Moreover, the students are given an introduction to Python programming, by letting them design the basic user interface to control the robot movements, as well as showing the HTTP camera input from the RobotCamWeb application.

The Level 2 learning process is organised in the following steps:

1. *Introduction to Python Script Programming*: Basic concepts about script programming, such as the editor, interpreter, importing libraries and using conditionals and loops, as well as variables and functions.
2. *Installing Python and OpenCV Library*: Solving the technical aspects of installing all the required software and libraries to solve the implementation of a Python-based user interface for the MiniCERNBot platform.
3. *Desktop Computer local camera monitoring*: Exercise to use OpenCV library in order to get the local computer camera preview in a desktop window (see Listing 1).
4. *Remote Android Camera Monitoring*: Implementing a Windows-based application that shows the camera preview of the remote android RobotCamWeb application, allowing the application of simple transformation to the acquired pictures, such as resizing and rotation (see Listing 2).
5. *MiniCERNBot Teleoperation with Onboard Android device*: Having the RobotCamWeb application installed and its Android device deployed on the robot, this exercise lets the student implement a simple teleoperation application that gets the camera feedback and provides access to the robot state and movements.

Listing 1 **Desktop Computer local camera monitoring template.**

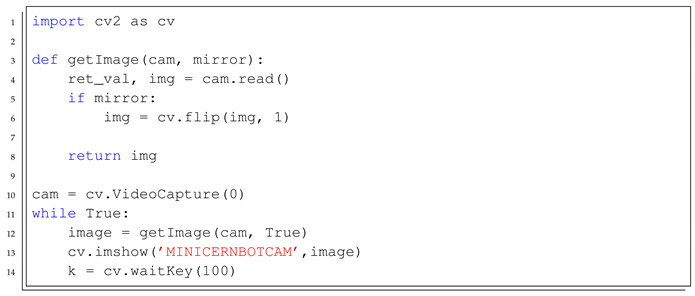

Listing 2 **Example of a Python template script to monitor the onboard Android device camera.**

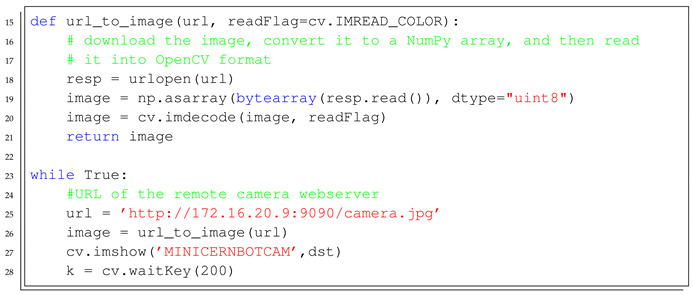

### 2.5. Computer Engineering Level 3 (Object-Oriented Programming)

Engineering and Master’s students are given the opportunity to learn and practise real problem-solving using Android Programming with Java Object-oriented language. In fact, two official courses at the Jaume I University are using the MiniCERNBot tool in the lab, (1) <<Networks and Mobile Devices>> at fourth course of Computer Engineering degree, and (2) <<Remote Control>> subject at the Intelligent Systems Master.

**Figure 18:**
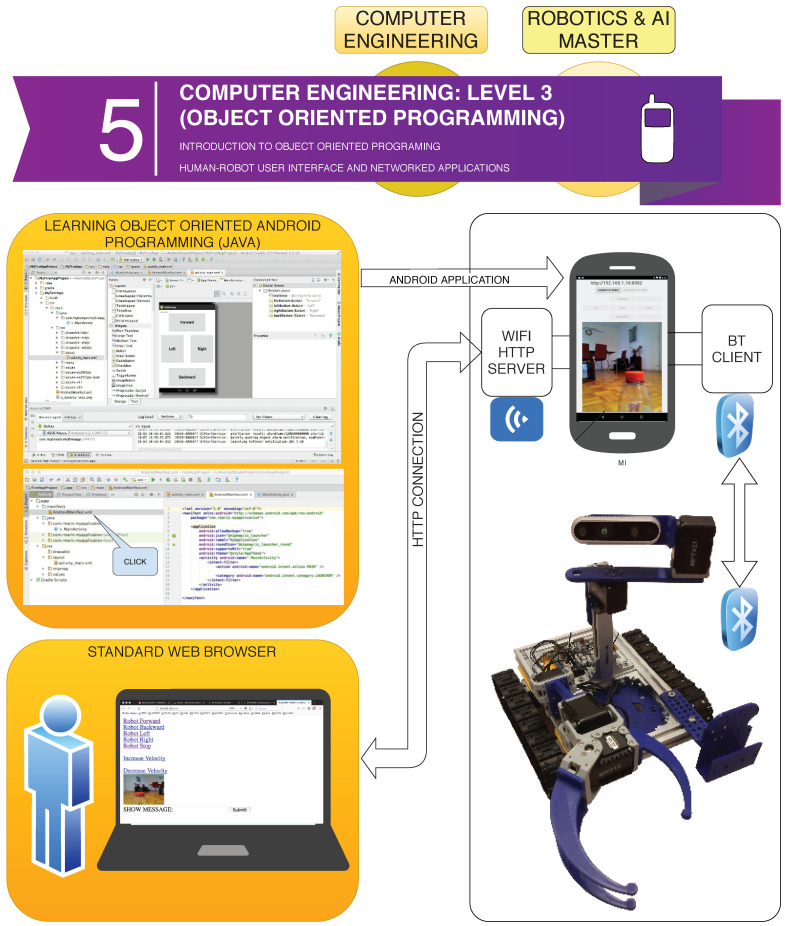
Software architecture to face the Level 3 learning path (i.e., Object-oriented Programming).

As can be seen in Figure 18, the students use as learning tool, the <<Android Studio Framework>>, which enables them to design low-level applications on Android devices, by using sophisticated API’s such as the Bluetooth and Wifi connections. In fact, the objective of the course is to design an Android Application that connects to the robot via Bluetooth in order to perform motor movements and sensor readings, as well as letting remote web browsers to access the mobile phone cameras and robot commands via Web (i.e., HTTP).

**Figure 19:**
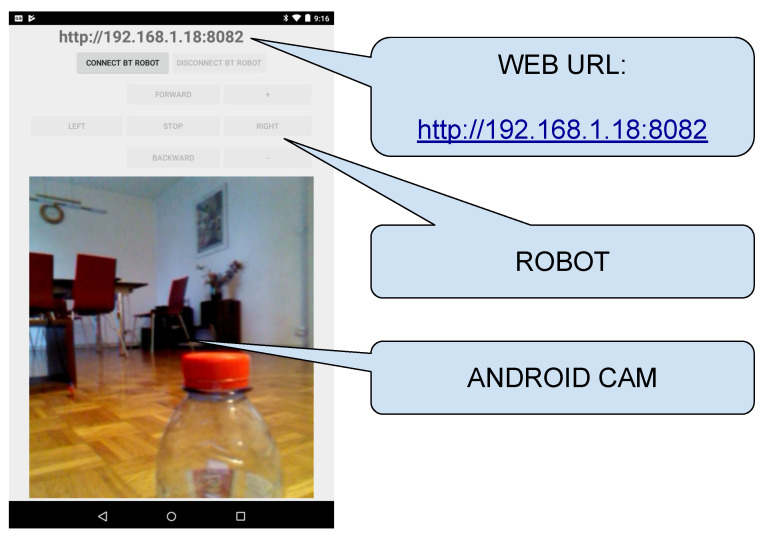
Android Application designed by engineering students, visualizing the mobile phone camera preview, buttons to control the robot via bluetooth, and showing the URL to let a web browser connect to this application and control the robot via web.

**Figure 20:**
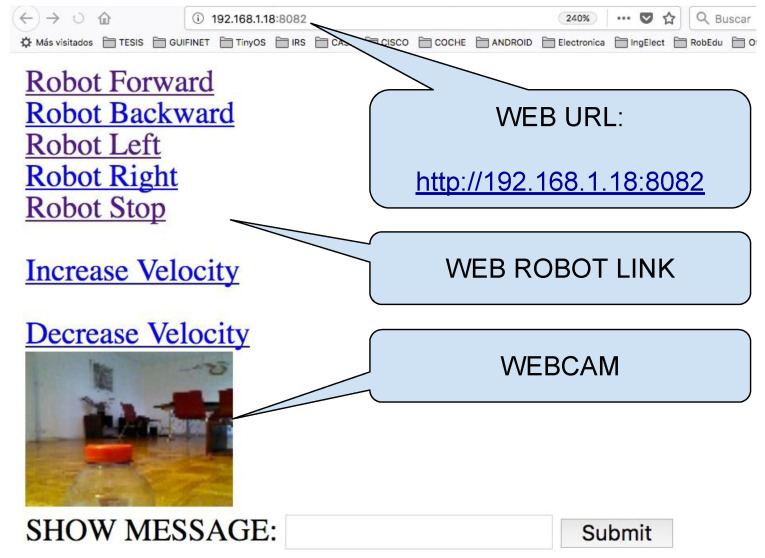
Web user interface to the remote robot, by showing an HTML file that is served from the onboard Android device Application running at IP (192.168.1.18) and port (8082), by using the URL address (http://192.168.1.18:8082 (accessed on 18 January 2021)).

The course is organised in the following way:

1. *Introduction to Java*: First steps on Java object-oriented programming language, studying classes declaration, functions, variables, and architectures, such as desktop, web, and Android applications.
2. *Introduction to Android Studio*: Explaining the structure of the Android Studio Programming Platform, understanding the basic steps to get an Android Application installed in the mobile phone.
3. *Bluetooth Connection*: Study the Bluetooth API that allows the Android implementation of Bluetooth clients and servers. In this step, the students have to implement a client to control the Minicernbot platform.
4. *HTTP Web Cam*: In this exercise, the students learn to implement an HTTP server on the mobile phone, which provides a web page to control the robot from a remote web browser. It allows also to get the Android Mobile Phone camera with a simple HTTP command (see Figure 19 and Figure 20).
5. *Proposed Projects*: The students are motivated to go further on the study of Android Programming using Java, by creating new related applications that might be of good use for society.

In Listing 3 and Listing 4, the Java and HTML templates to start the implementation of the Android application are detailed.

### 2.6. Applying the Solution

All the students that are involved in the CERN education program on robotics (i.e., high-school, engineering and Master’s) use their respective tools in order to solve the proposed missions on the antimatter factory mockup panel.

The importance of this step is crucial, as it gives students the learning focus, so that they find a real utility of the acquired new learning results. The facility to deploy the MiniCERNBot platform and control the movements gives an extra level of motivation, so that this learning step gives them feedback in order to better learn the previous tutorials.

Listing 3 **Example template to start the implementation of the Java object-oriented HTTPWeb Server on the Android Mobile Phone.**

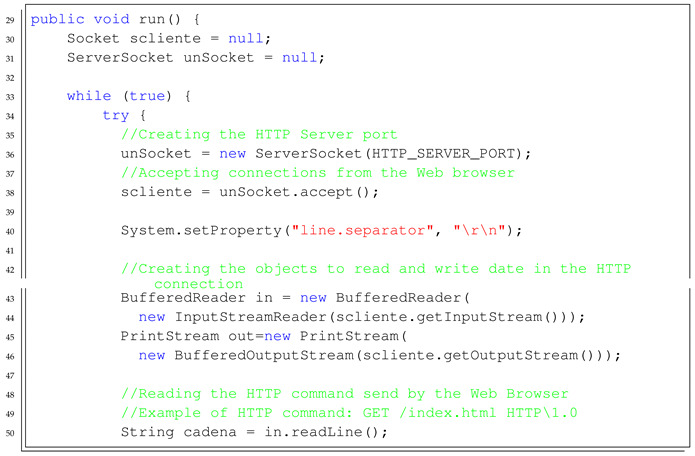

Listing 4 **Example template to start the implementation of the HTML file served by the HTTP Web Server on the Android Mobile Phone.**

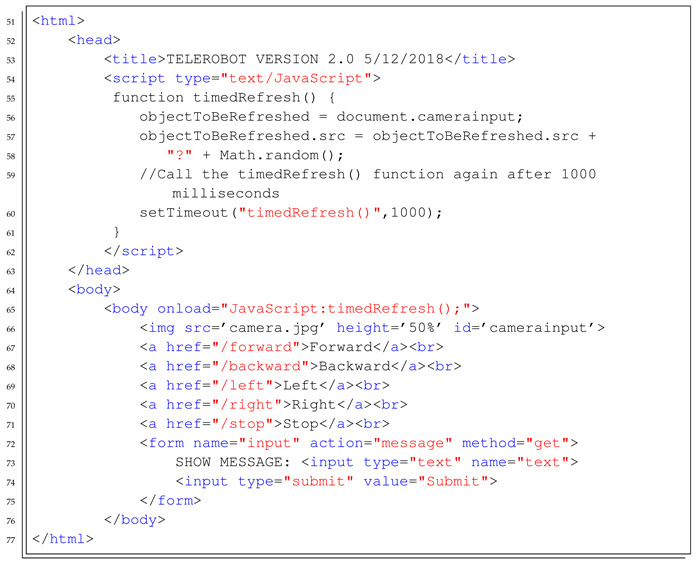

**Figure 21:**
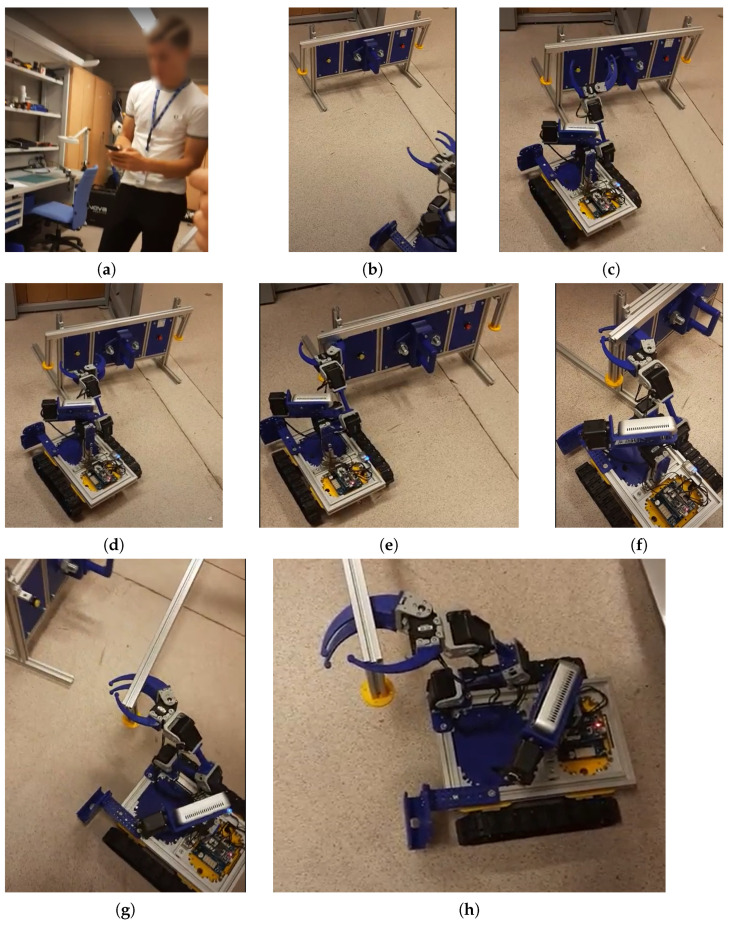
Comparison of steady state results (**a**) student teleoperating the robot to accomplish the first mission; (**b**) robot approaching panel; (**c**) student preparing arm to get the bar; (**d**) student preparing the approach to the bar; (**e**) closing the griper; (**f**) bar taken; (**g**) taking the bar back to the base; (**h**) bar recovered, mission completed.

In Figure 21, an example of solution applied by a student is given, where the student was able to solve missions of pressing the buttons and recovering the pipe, with the help of a manual support on the other side. This experiment gave the students the ideas to solve the next missions, such as unscrew, uncover, recover the target and pipe transport in a cooperative manner.

**Figure 22:**
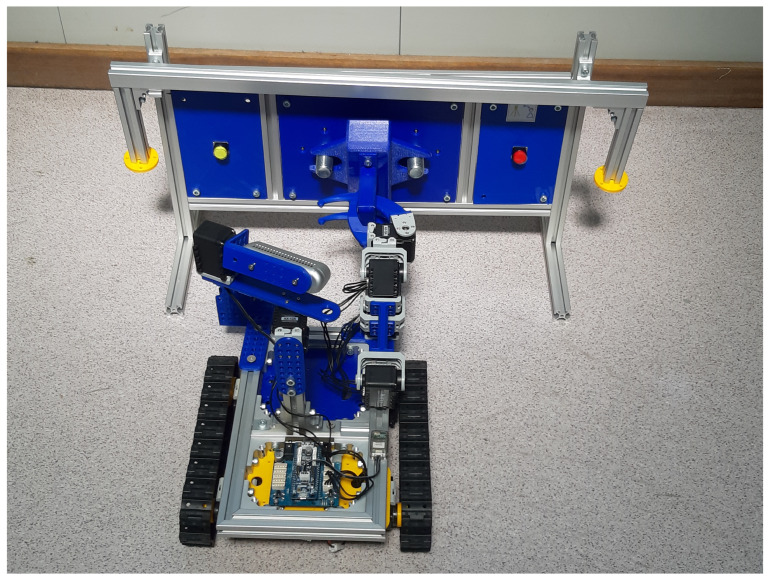
Students uncovering the antimatter factory panel mockup shield.

In Figure 22, the exercise of a student can appreciated while uncovering the mockup panel, in order to be able to recover the pretended-radioactive source.

### 2.7. Advanced Applications

The students are also given the possibility to develop more advanced applications, especially for those who spend more than one week in the lab, and have an engineering level equivalent to a Master’s, so that they have the opportunity to further learn the proposed tools.

The proposed missions are the following:

- *Mission 4: Cooperative Grasping*: This mission consists of helping the student to enhance the user interface in order to control two MiniCERNBot robots at a time. The robots should be able to approach the pipe handles and perform the grasping in a synchronised way.
- *Mission 5: Cooperative Transport*: Once the pipe has been grasped by the two robots, they have to bring it to the base. For this mission, they realize the necessity to use omni-directional wheels instead of tracks, in order to better have a wider range of movements the robot can perform.
- *Mission 6: Semiautonomous vision-based control* At this point, the students realise that some of the interventions can be performed automatically by the robots, under the supervision of the user, who is able to launch the missions in a more supervised manner. For this, they are offered the possibility to use the already existing vision-tracking algorithms developed at the CERN Robotic Framework, via a web Jupyter interface to design the corresponding python scripts, as explained in the next subsection.

#### Semi-Autonomous Vision-Based Control

A well studied and structured Python module has been developed and integrated in Jupyter [29], which runs from the client by using the camera installed on the robot (server) platform.

Such a module is fully written in C++ and OpenCV-based [30], which implements a set of static methods providing different solutions so that students can address every possible issue they could face by applying a range of strategies for overcoming successfully, fulfilling the necessities on each task, in a friendly manner, without the need for knowing the inner workings of either the computer vision algorithms or the cameras installed on the platform. Such a module offers to the students a soft introduction to computer vision, enhancing their training experience, and showing the power of integrating vision on the algorithms for robotics.

The module creates a singleton instance which generates a multi-thread system to treat most of the utilities in parallel with the aim to allow for the students to launch as many strategies as they decide to use. Each utility is well described in the help command (help(MCBV)) of the module, where the users can not only read the behaviour of tools provided, but also learn what they are usually used for. These utilities are listed below:

- *Launch the camera*: running on the back-end, allows the users to visualise the frames from the server just by tapping *launchTheCamera(MCBV)*. The frame rate is unaffected by the image processing (due to the parallelism) with the aim to bring the best image flow possible.
- *Start the tracking*: as the camera is launched, it is also working in a parallel thread. It tracks a Region Of Interest (ROI) chosen by the students from the frame. It offers either the possibility of drawing a square to let the students know the performance of the tracking (if the video s being displayed), or send a request to get the error when it needs. The last one allows for reducing the memory during the process.
- *Get the Error*: as mentioned above, this method gives the current distance (in pixels) from the centre of the ROI with respect to the centre of the frame either whether or not the image is displayed.
- *Colour filtering*: either by parameters (set of colours predefined) as sliding bars, the students are able to isolate the targets. With this, we not only get students to learn how useful the use of colour discrimination is in image processing, but they can also experience this by themselves with the enlargement or reduction of the RGB components.
- *Computer Vision*: Classic computer vision algorithms such as erosion, dilatation, histograms equalisation, and so on, which inserts students into the basic tools of image pre-processing, fulfilling the main goal of the module.

To run this module, the students have to import the vision module into their Jupyter notebooks (Jupyter client), and interact with the robotic platform by connecting to the Jupyter server which is running onto the robot side. The communication with the camera hosted in the robot is established via HTTP.

All of this allows students to work as a team either by sharing a computer, or on different workstations, whereby each can develop their own solutions to later pool them to achieve the best option to overcome the assigned tasks.

### 2.8. Learning Paths Summary and Tools

In summary, the experimented and described learning paths allow the student to get expertise on the tools presented in Table 1.

## 3. MINICERNBOT Platform Description

### 3.1. Architecture

In this section, the architecture of the robot is explained in detail. The MiniCERNBot is highly inspired on the CERNBot platform, a modular system for remote robotic operation in hazardous environments. Modularity is also a key concept for the MiniCERNBot robot, as it is for the CERNBot one, which is able to be reconfigured in hardware and software in order to face unexpected and sometimes urgent robotic interventions at CERN scientific facilities.

**Figure 23:**
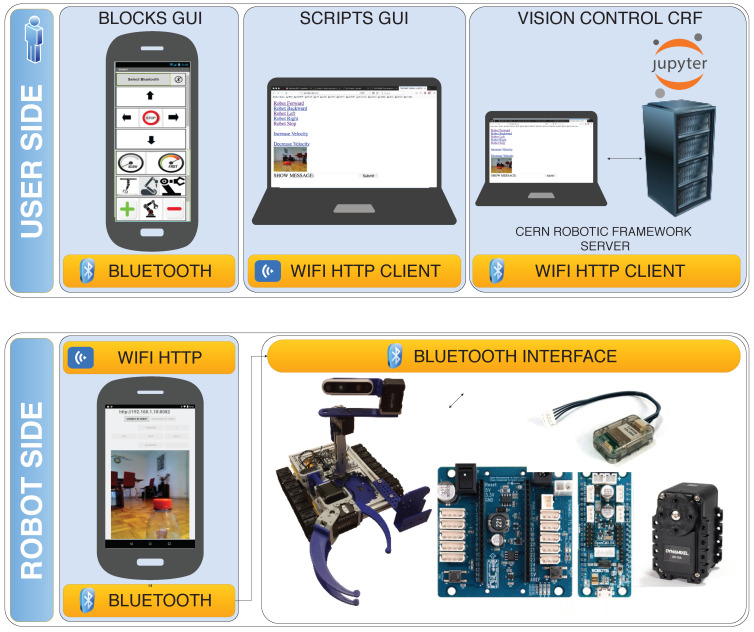
MiniCERNBot educational robotic platform software architecture.

In Figure 23, the software architecture of the educational platform and activities are presented.

First of all, the architecture is organised in two main blocks: (1) the robot side, which includes the Bluetooth operated robot hardware, and the onboard android Wifi controller, which acts as a relay between the client-side HTTP Requests, the mobile phone sensors (e.g., magnetometer, accelerators and cameras), the on-head 3D RealSense Camera, and the robot sensors/actuators; and (2) the user side, which allows different ways of designing user interfaces to the remote robot.

The robot electronics uses the following components:

1. *AX12A and AX12W motors*: A set of ten dynamixel AX12 motors, connected in chain to the micro-controller. It uses four motors for the base, two for the head holding a 3D camera, and four motors for the arm. The robot has been tested successfully with none, one and two arms.
2. *Robotis OpenCM9 and Shield 485 microcontroller*: It allows for getting control of the motors by using a bluetooth server firmware implemented in C through the Arduino IDE software.
3. *Robotis OpenCM9 microcontroller and OpenCM485 Expansion Board*: It allows for getting control of the motors via Bluetooth by using a C firmware based on Arduino IDE.
4. *Bluetooth Adapter*: Microcontroller bluetooth adapter to enable a serial connection to the robot in a wireless form.
5. *Sensors*: Sensors to be attached to the Dynamixel bus and the inputs of the OpenCM microcontroller.

Figure 23 represented the blocks that can be developed by the students as a learning path, including the Blocks GUI, Scripts GUI, Vision Control CERN Robotic Framework, and the robot-side on-board the Android HTTP controller. These blocks have been explained in detail in the previous section.

### 3.2. Mechanical Design

The aim of the mechanical design of the MiniCERNBot platform is to allow the modular attachment of motors, sensors and components (e.g., arm, head) to aluminium frames, in order to let the students adapt the robot configuration to the current mission need. In addition, it is very important to have a robot that is simple to set up, having it in operation in just a few seconds.

**Figure 24:**
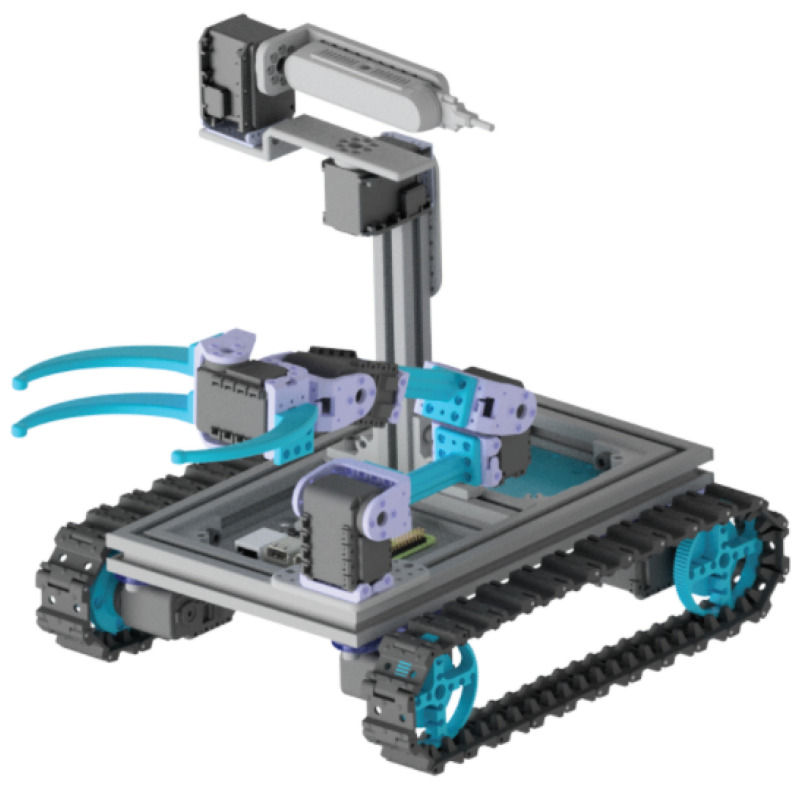
MiniCERNBot educational platform 3D design in its basic configuration.

As can be seen in Figure 24, the robot presents a basic configuration of a base with four motors, which can hold both trucks and omnidirectional wheels. In addition, it can hold robot arms in different configurations, as well as a head that provides the possibility to move a 3D camera in pan and tilt. The student is able to change the robot configuration in order to enhance the robot efficiency for a particular mission.

**Figure 25:**
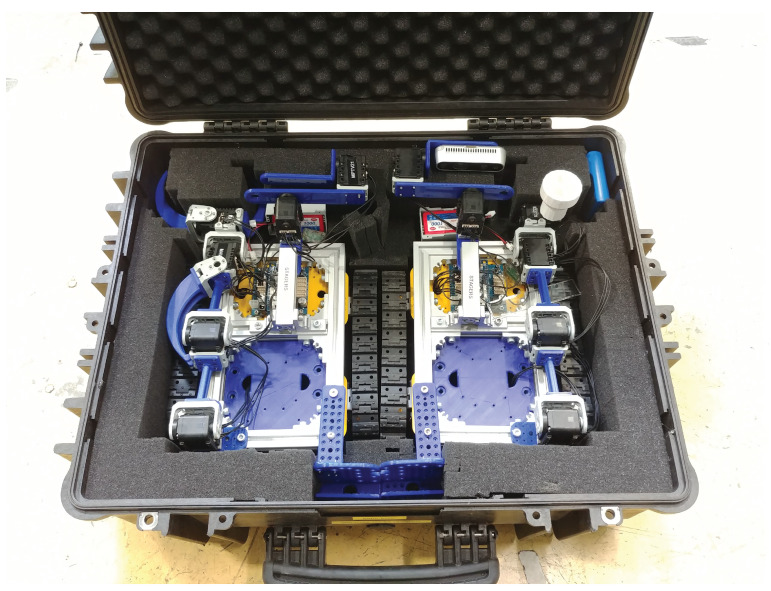
The MiniCernbot platforms in their storage case.

The MiniCERNBot platform is thought to be operated in twins, enabling the execution of cooperative interventions. In addition, they can be plied in a very simple way, by fitting the robot team in a single case, as shown in Figure 25.

In Table 2, some examples of mechanical parts are described. At the moment of writing, the MiniCERNBot platform includes a total of more than 50 mechanical parts, including also the accessories such as the exoskeleton and the master/slave arm.

### 3.3. On-Board Android Device

The MiniCERNbot educational platform uses as a main tool the ability to install the student Android device on-board. This possibility offers the students the facility to work at home and also in the lab with their own mobile phones, being able to use the robot for validation. The students are also provided with a mobile phone per robot, which can be used in case of necessity.

The provided Android device needs to serve HTTP requests for sensors (e.g., camera pictures) and robot commands, at an efficient manner, so the selected device tries to fit this requirement.

In fact, the performance experiments presented in the next section are using a Realme RMX1971EEA Android phone (Realme, Shenzhen, China), with the characteristics explained below.

In order to benchmark the Android device specifications, the CPU-z [31], GeekBench [32], and Android AI Bench applications have been used [33], so that the results can be better compared in further experiments. The specification of the Android device according to the benchmarks can be seen in Table 3.

## 4. Results

The results presented in this section focus on three main experiments, (1) robot modularity, (2) software architecture network analysis, and students’ opinions. In addition, this section concludes with a video demonstration of one of the robot experiments performed by the students (i.e., medical application). The robot modularity experiment tries to demonstrate that the system motivates and allows students to reconfigure the MiniCERNBot platform to solve different kind of projects. In addition, the software architecture network analysis allows for studying if the current architecture is efficient enough for academic purposes. The third experiment, students’ opinions, permit better understanding of the degree of motivation of the students, so that further steps can be taken in order to improve the system and enhance their learning experience.

### 4.1. Robot Modularity

Operations in hazardous environments need the integration of multidisciplinary sciences, and mechanical engineering is one of the main aspects to focus on. In fact, the CERNBot platform modularity has demonstrated to be very useful on adapting the robot to different configurations, so that it can reach a higher number of places in a safer manner.

In this experiment, a set of projects performed by high-school and Master’s students using the modularity feature are presented.

#### 4.1.1. Dual-Arm/Enhanced Gripper/Omni-Wheels/Omni Camera

**Figure 26:**
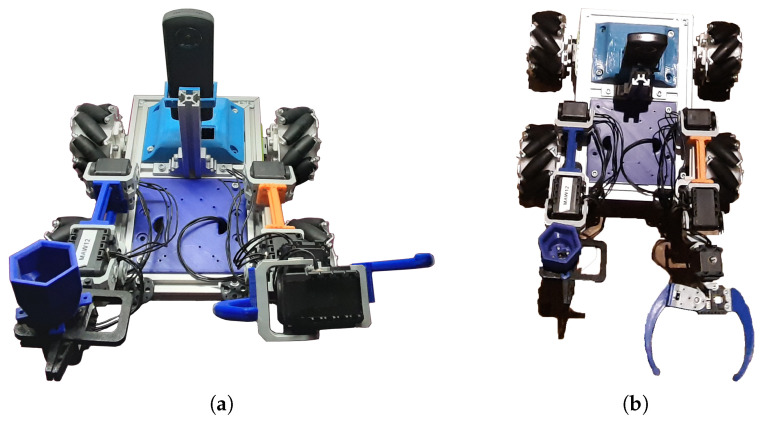
MiniCernBot reconfigured to have dual-arms, omnidirectional wheels, multipurpose right-hand, and 360° camera. (**a**) Frontal view, (**b**) top view.

Students were able to enhance the robot by providing a dual-arm configuration, including an enhanced gripper in the right arm, which can act in two ways, first as a single parallel gripper to pick up objects, and second as a screwing tool. In addition, in order to enhance the robot movements and be able to better intervene in the panel, the tracks were substituted by omnidirectional wheels (see Figure 26). Besides this, the pan-tilt head was substituted by a 360° omnidirectional camera. The new robot configuration has been very successful and one of the most interesting aspects is the collaboration between a high-school and Master’s student to get it ready for operation.

#### 4.1.2. Master–Slave Arm Project

**Figure 27:**
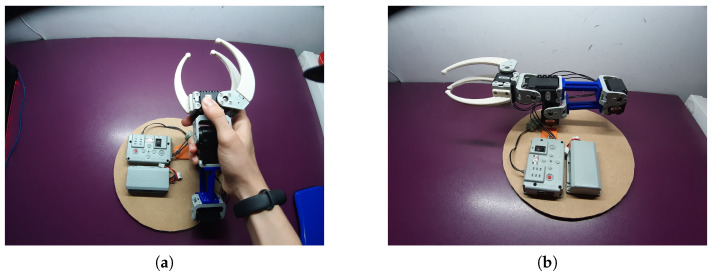
Student Project to implement a Master–Slave system to control the robot arm at a distance. (**a**) Frontal view, (**b**) side view.

As seen in Figure 27, students developed a master arm prototype in order to be able to remotely control the robot manipulation in a position master/slave manner. They could experience the different kind of teleoperated systems, better understanding the high-fidelity manipulation movements that a master device provides.

#### 4.1.3. Exoskeleton for Improved Manipulation

**Figure 28:**
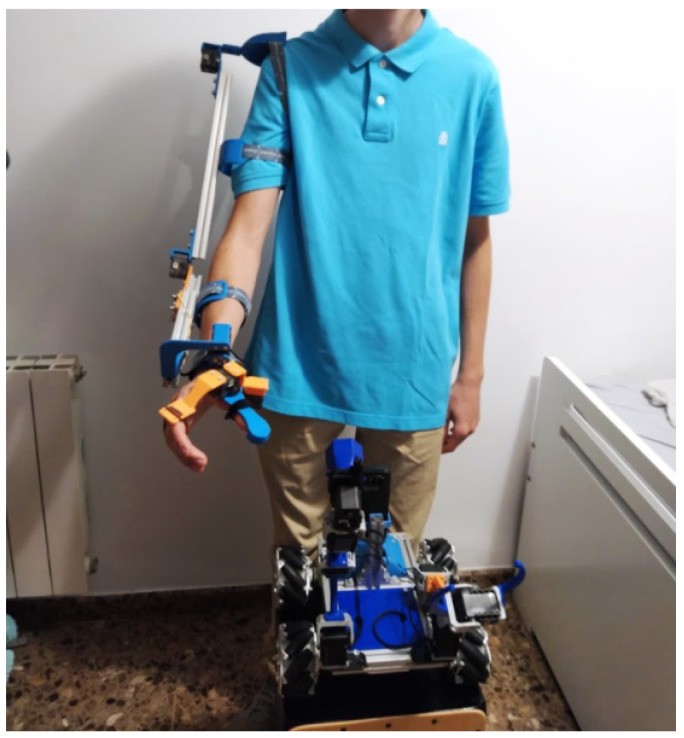
Exoskeleton designed by the student in order to remotely control the robot arm.

In Figure 28, we can appreciate the master–slave project extended, so that it is used as an exoskeleton. The level of motivation of the students with this project was very high, in terms of mechanical engineering. In addition, it is still under improvement, and the current focus is on the electronics integration.

#### 4.1.4. Transformer Designs

**Figure 29:**
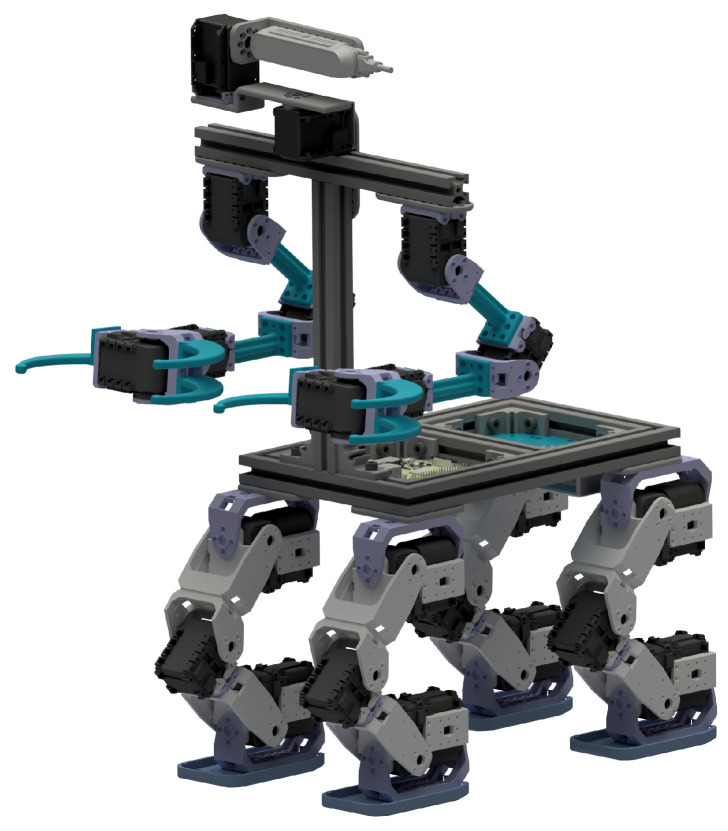
An alternative design with four legs like a centaur that a student designed for an environment with rocks.

**Figure 30:**
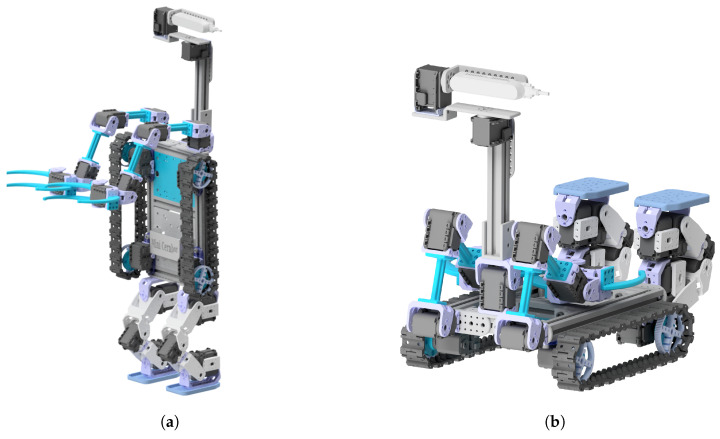
Transformer design that enables the robot to have two distinct configurations. (**a**) Transformer configuration to manipulate a target, (**b**) configuration to explore the environment.

Thanks to the MiniCernBot’s modularity, once students are finished with the course, they are able to change the designs of the robot, making them improve their designing skills, and making them think creatively to provide interesting configurations for approaching different tasks or environments (see Figure 29 and Figure 30).

#### 4.1.5. High-School Student Medical Design to Reduce the Spread of Covid-19

**Figure 31:**
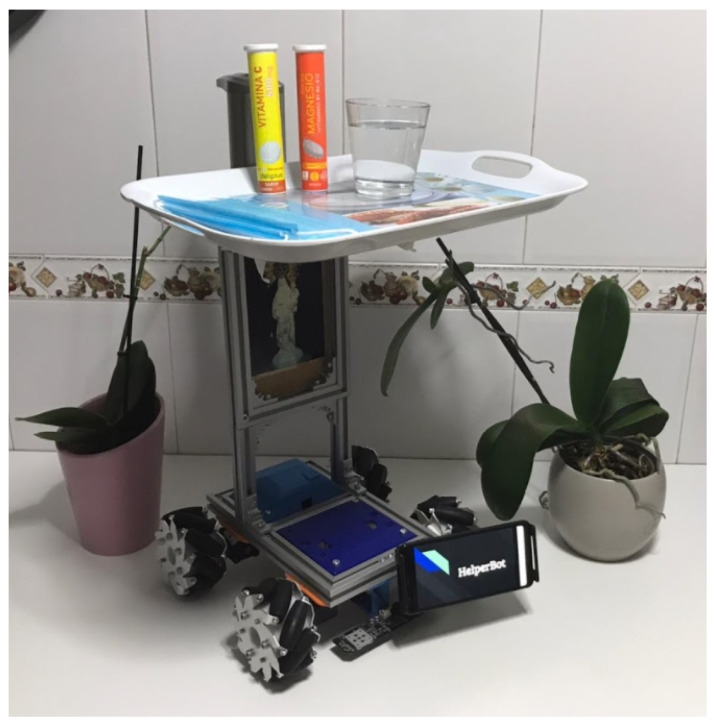
Helper Bot, MiniCernBot’s configuration for medicine applications.

During quarantine, a specific educational MiniCernBot configuration was designed by a student for a medical application, with the idea of reducing the spread of Covid-19 inside hospitals, making the interactions between the doctors and the patients safer (see Figure 31).

As it can be appreciated in Figure 31, the robot has an attached tray so that it can transport water, food or medicines to the patients, without the doctors having to get near the patient, improving safety, efficiency, and reducing medical equipment such as masks. The robot contains a cover to make its disinfection quicker, an automatic alcohol dispenser, omni-directional wheels to improve the mobility of the robot, a colour sensor allow following a circuit autonomously, and a phone attached to the robot, in order to establish communication with patients and give vision to the user.

While doing this project, the student improved drastically his designing skills as he designed all the new pieces such as the automatic alcohol dispenser attachment for the robot. He also further developed his problem-solving skills, as he had to confront different problems during the project. He learned about programming as he made a program to make the robot move autonomously through a circuit with the light sensor, and, finally, this project made him interested about the world of medical robotics, giving him a better idea of what to study at university.

### 4.2. Software Architecture Network Analysis

In this section, the software architecture is analysed from the network performance point of view, considering that using a mobile phone on-board provides a lot of possibilities in terms of education, but it is also necessary to study its effects in terms of computer performance.

**Figure 32:**
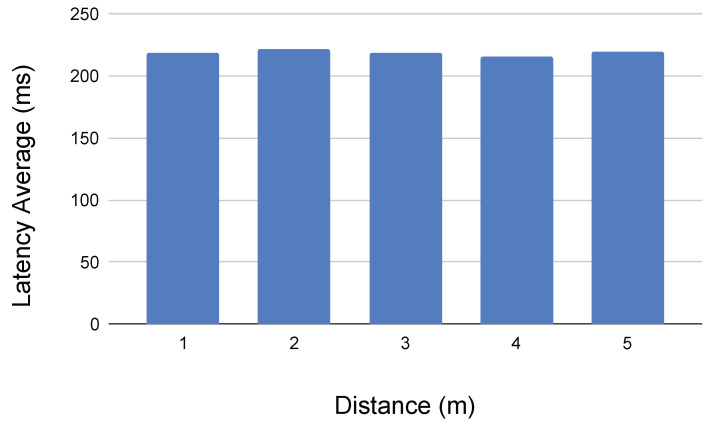
Latency results obtained by sending a <<get state>> request to the the robot via Bluetooth from the Mobile Android Device.

First of all, in Figure 32, the network latency between the robot controller and the Android device via Bluetooth is shown.

This latency is the result of requesting via bluetooth the state (i.e., position and velocities) of all the motors in the bus. It means that the microcontroller receives the bluetooth request, then it sends a command to the Dynamixel bus in order to read the memory values at every motor and sensor, and then it returns a JSON string with the state values via the Bluetooth adapter, to be received at the Android Blocks application. The latency is calculated from the client mobile phone.

We can appreciate that the signal is very stable when teleoperating the robot in the same laboratory (i.e., less than 5 m), being able to get the real motors and sensors position in the client side at a rate of five times per second approximately.

**Figure 33:**
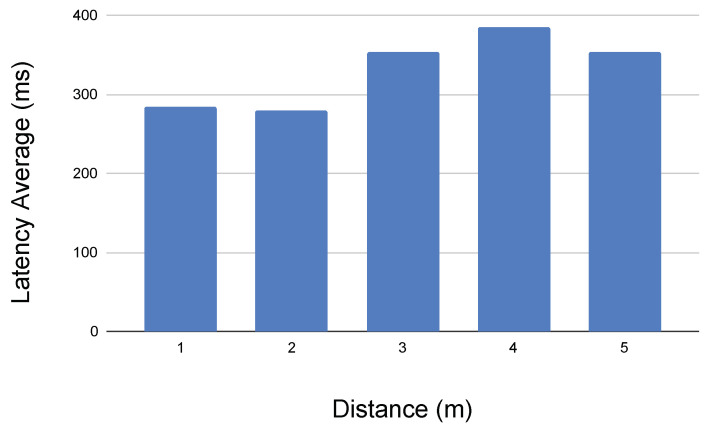
Latency results obtained by teleoperating the robot with a scripting GUI via the on-board HTTP Android device.

In Figure 33, the latency provided by a Python script user interface interacting with the robot via the onboard Android HTTP application is presented. As in the previous example, the latency is calculated in the Python user interface, which requests via HTTP to the Android Phone the current state of all the motors in the Dynamixel bus. It includes the HTTP management and its interaction with the microcontroller via Bluetooth.

First of all, we can conclude from the previous results that the latency varies slightly from distance due, according to our comprehension, to the lack of automatic gain applied in the GUI Wifi adapter, when the Radio Signal Straight Indicator is reduced. It means that the signal quality is reduced with distance, increasing also the effect of reflections and the necessity of the higher protocols to resend the packets.

In addition, we can appreciate that the HTTP server implemented on the on-board Android device is adding a significant amount of delay to the communication. Further efforts will focus on enhancing the performance of this server. At the moment of writing, its software design uses two multi-thread services, one that takes care of the graphical interface plus the Bluetooth connection to the robot, and a second that implements the web server. It means that threads have to communicate via pipes, which increases the delay significantly.

Moreover, the current version of the HTTP Server Android Application implements the HTTP 1.0 protocol, which closes the connection after every request. It means that, in order to send a command to the robot, the connection has to be established (i.e., hand-shaking with three packets), the information has to be sent and acknowledged (i.e., four packets), and finally the connection has to be closed (i.e., three packets). The next implementation of HTTP 1.1 version will permit the client to ask the server to keep the connection alive, which will enhance the overall system performance during the educational exercises.

**Figure 34:**
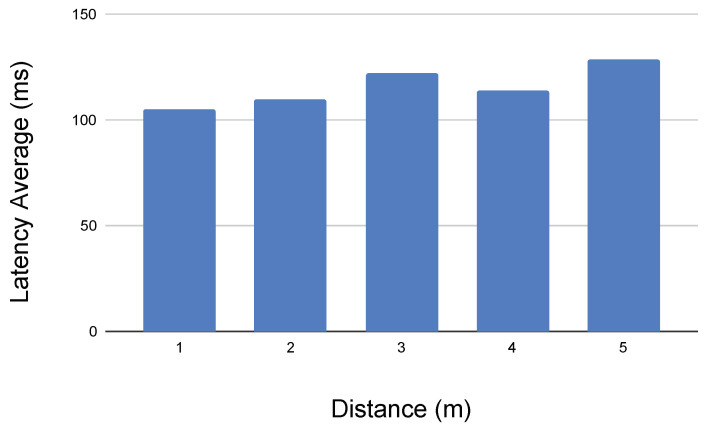
Latency results obtained when requesting the onboard Android Device camera picture (i.e., 640 × 480 pixels JPEG color).

In Figure 34, the latency obtained when getting the current on-board Android phone camera picture via HTTP to a computer Python script is presented. Results show that the image feedback in the user interface can be offered at a rate of 10 frames per second, resolution of 640 × 480 in color, which is enough for educational purposes.

We can conclude that the HTTP connection using Android, as it is implemented, is very flexible and useful for the academic purposes, and requires a much better enhancement in order to face bigger challenges. For students, using an Android phone as a robot brain is very convenient, as can be seen in the next section.

### 4.3. Students’ Opinions

In order to better assess the motivation and learning of the students, the students’ opinion has instead been requested on the level of both complexity and satisfaction, for the time to carry out every proposal task, for the overall experience during their internship period.

**Figure 35:**
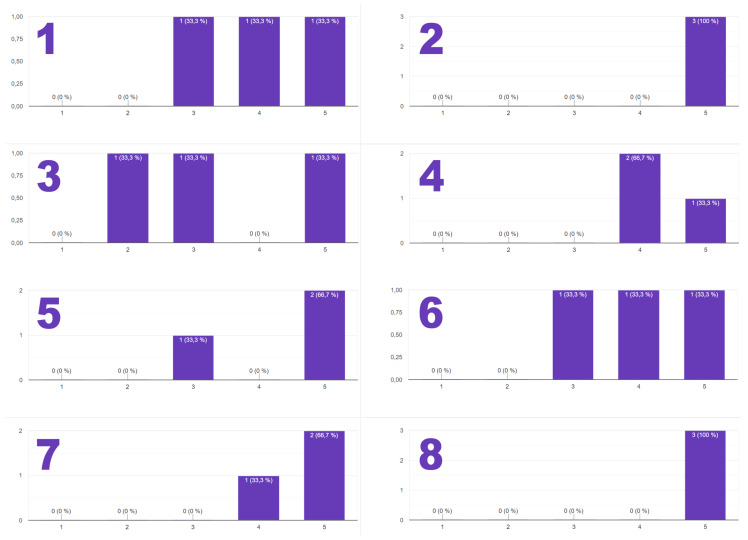
Success rate of the tasks: (1) Assembly (motors, parts, aluminium, etc.), (2) Android User Interface Application (MIT APP Inventor), (3) Semi-autonomous Vision-based Python Control, (4) Robotic Mission (pressing the button), (5) Robotic Mission (unscrew), (6) Robotic Mission (recover shield), (7) Robotic Mission (recover radioactive source), (8) Overall Level of Satisfaction.

**Figure 36:**
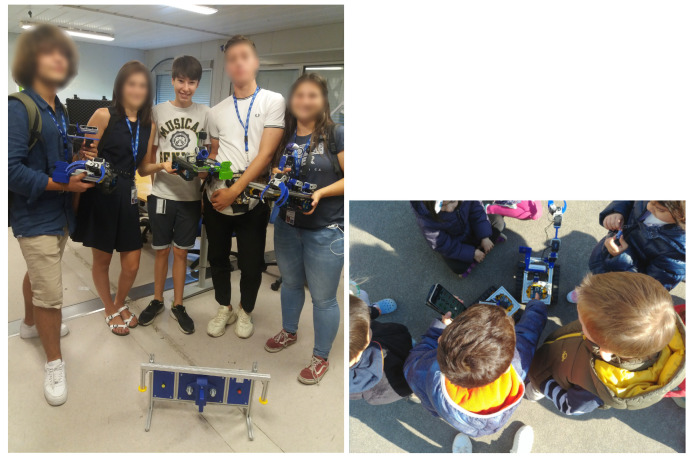
Final test with students: (**1-left**) High-School Summer Stagers; (**2-right**) Children at CERN’s kindergarten interacting with the educational robots developed on this project.

As evidence, in Figure 35, where the level of success of each task is presented, most of the tasks were resolved with a high score percentage, providing a maximum level of satisfaction to the students’ teams (see Figure 36).

In addition, the students were requested to provide an opinion about the internship period, as well as to provide a new proposal with the aim of bringing improvements to the program for future students. Some of these opinions are listed below:

- I liked this stage because it didn’t have all the things like: reunions, staying for more than 1 h listening to something you aren’t interested in. I liked the thing that i didn’t had only one "maitre de stage" and that everyone taught me what they knew without being too complicated for my age/capacity of comprehension. With this experience i could finally train my pronunciation in English because few people were french and they were mostly Italian and Spanish.
- The Robotic Stage was fun and entertaining. I learned lots of new things and skills both during the creation of the project for the "unscrew" tool and during the assembly and testing. If I had to change something, I would provide a more detailed set of instructions on how to operate the 3D camera through the GUI and how to create the semi-autonomous vision system using PY (which was given a 3 neutral score because it was not covered during my stage and was not included in the documentations). Overall, I feel really satisfied and happy about my experience at CERN. The environment was friendly and chill. I would definitely do it again.

In addition, as can be seen in Figure 36-right, several education robotic demos have been performed at the CERN’s kindergarten with the purpose of bringing robotics to the little ones, showing a high degree of motivation in further STEM related studies.

### 4.4. Videos

In this section, a video of the MiniCERNBot platform is shown, adapted in order to simulate a medical intervention to avoid COVID exposure of the doctors. The project was done by a high-school student, enormously motivated on the design of the system.

- *Medical Application:*https://drive.google.com/file/d/13ZMwjQ8qd44wHDl5eavhAPKGtDnOYRwd/view?usp=sharing (accessed on 18 January 2021)

## 5. Conclusions and Further Work

This paper describes the problem-solving strategy used at CERN Robotics Lab in order to let visiting students to have a better understanding of the research activities, as well as the way to solve them technically with the help of robots. The main goal is using technology for the peace and safety of the users, working jointly in order to progress in science and social applications.

The MiniCERNBot platform and its learning path have resulted in being very positive in terms of motivation, giving students a realistic example of the activities performed at the Lab, in an educational and practical manner. All of the students gave a very positive feedback and showed their gratitude to the whole team, encouraging to go further in order to offer improved learning activities in the future.

Aspects, such as team organisation, brainstorming, multidisciplinary solutions, and balance between guided tutorials and project-based learning sessions, have demonstrated to be very important in the students’ learning experience. We have also realised the importance of letting students base their training on real references, such as the robotic intervention in the antimatter factory, which result in higher motivation. It is in fact this motivation that has resulted, in the authors’ opinion, in being the most interesting aspect of this learning MiniCERNBot educational experience.

In addition, it is worth mentioning that, for the experiment presented in this paper, it was not possible to obtain real data of the degree of influence of the educational experience on later high-school students’ vocations. On the other hand, a pilot experiment was performed at the Jaume I University, in collaboration with CERN, where the MINICERNBot robot was used in two seminars for high-school students, in order to promote Computer Engineering studies. Ten students out of twenty-five (20%) followed further Computer Engineering studies. Moreover, from twenty-two Master’s students that followed the educational experiment, two of them joined the doctorate program for further studies on field robotics (i.e., radiation and underwater).

Moreover, the system can be improved in several aspects, in order to provide better learning experiences. In fact, taking into account the current COVID-19 situation, a realistic 3D simulation system, which can be accessed via the web, letting students work in a collaborative way, and compare their results with other teams, can help enormously in order to increase the value of the system. Besides this, going beyond simulations and letting students solve the problems remotely, with the real robots via web, would be even more challenging. Further work will focus on this direction, enhancing the way students communicate with simulations and real robots, via the web, in a collaborative online learning experience. In addition, the learning paths presented in the paper can be enhanced by using the tool in more disciplines and educational levels. Some preliminary experiments have been started with this aim, by using the MINICERNbot platform to introduce advanced research robotics to PhD students, as a desktop reconfigurable robotic platform, in order to test the experiments before applying them to more realistic scenarios.

## Figures and Tables

**Table 1:** Relation between learning paths and experimented tools.

Learning Paths Summary and Tools			
Learning Activity	High-School Path	Computer Engineering Path	Master on Robotics Path
1. Study the Problem and Solution in Groups	Multidisciplinar team	Multidisciplinar team	Multidisciplinar team
2. Mechanical Engineering	Tinkercad	Tinkercad or onShape	Tinkercad, onShape or Autodesk Inventor
3. Computer Engineering Level 1 (Blocks)	MIT App Inventor		
4. Computer Engineering Level 2 (Scripts)		Python	Python
5. Computer Engineering Level 3 (Object-Oriented Programming)		Android Studio	Android Studio
6. Applying the Solution	Minicernbot and Antimatter Mockup	Minicernbot and Antimatter Mockup	Minicernbot and Antimatter Mockup
7. Advanced Applications			Two Minicernbot robots and Antimatter Mockup. Vision library of the CERN Robotic Framework

**Table 2:** Examples of mechanical parts available at the MiniCERNBot robot.

Mechanical Part	Description
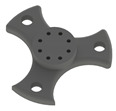	Piece for attachment of the dynamixel AX12 motors to the omnidirectional wheels
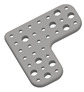	Parts that allow the attachment of an arm in one of the corners of the MiniCERNBot Base platform
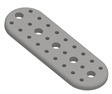	Adapter for attached dynamixel AX12 motors to a generic aluminium frame. It can be used to attach tools and also wheels to the base

**Table 3:** On-board Android Device Benchmarking results using CPU-Z, Geekbench, and AI Benchmark application for Android.

On-Board Android Device Benchmark Results	
Feature	Device Specifications
Mobile Phone Model	Realme RMX1971 (RMX1971EEA)
Operation System	Android 10 (API 29) Build RMX1971_11_C.08
Architecture	Kryo 385 revision r7p12
Kernel	Architecture aarch64 Kernel Version 4.9.186-perf+ (1601266300)
Processor	Qualcomm Snapdragon 710 2,30 GHz (Cores 8)
RAM	7659 MB
Cores	Cluster 1 6 Cores 1.71 GHz; Cluster 2 2 Cores 2.30 GHz
GPU	Adreno (TM) 616
Geekbench Score	411 (Single-Core) 1548 (Multi-Core Score)
Geekbench Crypto Score	694
Geekbench Integer Score	403
Geekbench Floating Point Score	381
Geekbench Camera score	380: 4.40 images/s Multicore 1172 13.6 images/s
Geekbench Machine Learning	194 7.50 images/s Multicore 597 23.1 Images/s
Geekbench Image Compression	448 21.2 Mpixels/s Multicore 1951 92.3 Mpixels/s
AI Benchmark Overall Result	33.2 (Good Performance)
AI Benchmark fp16 NN Speed	32.6%
AI Benchmark int8 nn-speed	54.4%
AI Benchmark fp16 Accuracy	54.8%
AI Benchmark int8 Accuracy	90.3%
AI Benchmark fp16 CPU	59.8%
AI Benchmark int8 CPU	26.7%

## Abbreviations

The following abbreviations are used in this manuscript:

CERN	European Organization for Nuclear Research
STEM	Science, Technology, Engineering and Mathematics
MRO	Mechatronics, Robotics and Operations
SMM	Survey, Mechatronics and Measurements
TIM	Train Inspection Monorail
GUI	Graphical User Interface
MIT	Massachusetts Institute of Technology
HRI	Human-Robot Interface
RSSI	Radio Signal Strength Indicator
HTTP	HyperText Transfer Protocol
URL	Uniform Resource Locator
API	Application Programming Interfaces
MCBV	MINICERNBot Vision
ROI	Region Of Interest
RGB	Red Green and Blue
IDE	Integrated Development Environment
JSON	JavaScript Object Notation
RS	Robot State
UJI	Universidad Jaume I
